# Participation of policy actors in the development of health policies in India and Nigeria and the implications for the role of evidence in policy-making

**DOI:** 10.1186/1472-6963-14-S2-P27

**Published:** 2014-07-07

**Authors:** Reinhard Huss, Mahua Das, Bassey Ebenso, Bindiya Rawat, Obinna Onwujekwe, Giuliano Russo, Lucie Blok, Putthasri Weerasak, Tolib Mirzoev

**Affiliations:** 1 Nuffield Centre for International Health and Development, Leeds Institute of Health Sciences, University of Leeds, Leeds, UK; 2 Association for Stimulating Know How, Gurgaon, India; 3 College of Medicine, University of Nigeria Enugu Campus, Enugu, Nigeria; 4 Instituto De Higiene E Medicina Tropical, Lisboa, Portugal; 5 Het Koninklijk Instituut Voor De Tropen, Amsterdam, The Netherlands; 6 Mahidol University, Bangkok, Thailand

## Background

Policy-making can be described as a complex decisionmaking process involving different actors engaged in negotiating their interests [1] and promoting different types of evidence. Policy informed by research evidence is widely seen as a good thing. However Monaghan [2] indicated that ‘Policy-makers may fish for evidence, select the beneficial bits and throw back those that are un-required’. This discretion in evidence and policy (EaP) processes and the selected participation of policy actors is inherently political. Our research analyses the participation of actors in EaP processes in India and Nigeria.

## Materials and methods

We examined three policies in each country: internationally-prominent; internationally-neglected and health systems policies. The study was guided by a conceptual framework linking EaP processes. Qualitative data was collected using documents review and in-depth interviews and analysed using framework approach.

## Results

We identified seven groups of actors involved in EaP processes with different and specific evidence preferences. The executive branch of government and civil servants were leading policy development, while legislators and judiciary appeared to be absent in both countries. Academics with limited power provided research evidence and participated in all policies except for tobacco control, where the tobacco industry was involved. Health workers were less visible in both countries. CSOs played a prominent role in India advocating for vulnerable people and contributing context-specific evidence. Development organisations played a powerful role providing expertise and resources for the production of evidence except for the policy on social activists in India. The media played no significant role in both countries except for the Indian AIDS policy.

## Conclusions

The participation of powerful actors in EaP processes can influence the contribution of evidence in policy-making. The involvement of diverse policy actors in EaP processes may ensure a wider array of evidence types, promote social learning and ultimately strengthen policy and practice.
